# An Address of Welcome to the American Dental Association, at Chicago

**Published:** 1865-08

**Authors:** 

**Affiliations:** Chairman of the Executive Committee


### AN ADDRESS OF WELCOME TO THE AMERICAN DENTAL ASSOCIATION AT CHICAGO.

BY DR. ALLPERT,

*Chairman of the Executive Committee*

MR. PRESIDENT,

*And Gentlemen of the American Dental Association:*

In bidding you welcome to the city of Chicago, on an occasion so interesting and auspicious to our Profession as the present, it may not be deemed inappropriate to the time and place of our meeting, to indulge in a brief retrospect of the past.

In the summer of 1859, twenty members of the Dental Profession met in convention at Niagara Falls, to consult together as to the expediency of forming a National Association upon the representative basis. All, I believe, who were present felt that if such an association could be formed, and should receive the sanction and co-operation of any considerable portion of the better class of practitioners, the best interests of the Profession would be promoted, and great good result to the public.

The number of state or local societies, then existing, to send delegates to the annual meetings of an association of this kind, was so very limited, that but few even of the small number present felt at all sanguine of the ultimate success of such an enterprise. But in view of the many great and and good results anticipated from such association, in case it should be crowned with success, and in the hope that the formation of local societies would be stimulated thereby, it was determined to take the initiatory steps for the organization, deferring final action until the following year.

At the appointed time, July 31st, 1860, *twenty-three* delegates only, of the various Dental societies and colleges then existing, met in the city of Washington, and the American Dental Association was organized, and entered upon its work. Five years only have passed, and from what was a small and doubtful beginning, by the steady and well-directed efforts of those who were instrumental in its formation, this association has become one of the most successful enterprises of the Profession, and one of the most useful and influential Dental societies in the world.

Of the professional standing of those who have attended as delegates, the annual meetings of this Association, and have been accustomed to take part in its proceedings, it is not necessary for me to speak. They are universally acknowledged as standing among the most scientific and successful operators of our time and country, and as belonging to the class *progressive.* The Essays and Discussions of the members of this Society have passed into the literature of our Profession and become a part of its history. In point of ability they have been by far the ablest and most instructive that have ever emanated from any body of dentists in our country, and will suffer little, if any, in comparison with the essays and discussions of the American Medical Association. Should this Association adjourn *sine die* to-day, and never hold another meeting, yet sufficient good has already been accomplished by it to fully vindicate the wisdom and foresight of those who first projected it, and have done the most to sustain it.

As we cast our mind's eye over the history of Dentistry, and see how, in the last forty years, it has risen from a tinkering, catch-penny calling to the dignity of a noble profession, in whose ranks may be found men of high moral and scientific culture, commanding alike the confidence and respect of the educated and refined, we can but attribute much of this progress and pleasing result to the influence of our various local and national associations. All of these associations have their influence for good, and are important, but none is so well calculated, in every respect, to allay that spirit of jealousy and distrust in each other—that fatal millstone around the neck of all selfish communities—none so well calculated to strengthen the bond of a common interest and brotherhood, that should bind the Profession of the East with the West, the North with that of the South, and make all to feel their mutual dependence upon each other, as a representative National Association. In it are embodied the principles that underlie the structure of our national government, which has demonstrated to the world, that for the protection of a community of interests, or for the development of resources, whether material or *mental*, no organization or government is so strong as that based upon the principles of a representative Republic.

You are all familiar with the advantages of association and combined labor in the various avocations of life, no matter whether it be in mental or physical labors. You assemble here to-day, as delegates and members from different parts of our extended land, and in the discussions here elicited you will find new illustrations of the old familiar truths that "knowledge is power," "union is strength," and "in a multitude of counselors there is wisdom." By the contact of mind with mind both will be strengthened, and embryo ideas and theories will be developed into full maturity. "There is a magnetism in such contact, full of creative energy. Flint and steel are passive in themselves, but clash them together and they give out fire, and brightness dazzles upon the sight. The positive and negative poles of a battery never come together without a *result.* By friction of different mental organizations together, an idea is evolved, a new law is discovered, a new creation is added to the wealth of knowledge, and the long-coming rays of a new truth, like those of a far-off star in the laboratory of heaven, reach and illumine the world."

Gentlemen, this Society was organized for a purpose. Its mission is not yet fulfilled. It has a great work to do—a destiny to accomplish. The men who were instrumental in its formation were not discouraged because it was so small and unpromising at first. They knew that in the Profession it had a strong and vigorous mother to nurse it, and that its growth was certain, but they did not expect it to mature so rapidly. Nor will those who are now engaged in it be so elated by its unexpected success as to allow it to sink under that supine and careless indifference, which so often follows prosperity. Its course will be onward and upward—its motto, Union and Progress.

As the scourge of war passes away from our country, and peace " with healing in her wings returns to bind up a nation's wounds," and those who have hitherto stood arrayed against each other in deadly conflict shall engage in the various pursuits of industrial activity; as that portion of our country which has been laid waste by the desolating hand of war shall be rebuilt, and the millions, North and South, in trust and harmony, shall once more labor to develop the untold treasure of our broad domain, we may look forward to a period of unexampled prosperity in the history of our country.

The rich cotton plantations and rice fields of the South, with the broad prairies and fertile valleys of the North, shall yield an abundant reward to the freemen's hand; the slave pen and human auction block shall give place to the church and the school-house; with human slavery, the source of all our troubles, forever extinguished from our land, honest pay shall be awarded for honest toil, and we may look for a season of prosperity such as the country has never known.

Wealth, education and refinement will become more general; and our Profession will be called upon to administer to the wants of a higher civilization. To ameliorate suffering and contribute to these wants should be our aim.

To be the better prepared to do this we should divest ourselves of all selfishness and vanity of opinion; and with uncovered heads drink of the fountain of knowledge, from whatever source it may flow. We should measure ourselves with ourselves, not to show that one man is stronger than his fellow, or to pull down those who have been more successful than ourselves, but to give just confidence to the timid, and to strengthen and raise up the weak. None should be ashamed to learn, or afraid to teach. Freely to give and freely to receive should be our object.

Some of you, before reaching home, will have traveled thousands of miles to attend this meeting. It may be you have come burdened with the rich treasures of experience and skill, to lay them upon the common altar of your Profession, for other's good; or, it may be you have come to drink from the fountain of thought which others have prepared for you. Here as ever, he who would freely receive should freely give. And here, too, the "widow's mite" is as acceptable as the rich man's treasure. Whether you have come to give or to receive, gentlemen, as Chairman of the Committee of Arrangements of the American Dental Association, I bid you welcome to the Garden City of the Lakes—to the Commercial Metropolis of the North-West.

Many of you visit to-day, perhaps for the first time, the city of Chicago. As you walk our streets, and see our magnificent and well-filled stores, our palatial residences, our splendid churches, our model schools and colleges, our grain elevators and warehouses—the largest in the world—our shipping, our railroads, branching out in every direction, our magnificent Chamber of Commerce with its massive and solid proportions, and this artistic and imposing opera building in which we meet; as you look around and see here the largest grain depot on the continent. a city stretching out for miles in every direction, with a population of two hundred thousand souls, it may not be amiss for me to state that it is not yet thirty years since Chicago was known only as a military post with a few Indian traders, who were supplied with the comforts of life from the East.

The very spot on which we are assembled to-day, in this splendid temple devoted to the fine arts, in the centre of this great commercial emporium, thirty years ago was but a hunting ground covered with prairie grass, through which the Indian pursued his game.

And yet last year our city sent forward to the seacoast and to European markets nearly fifty million bushels of grain, enough, if placed in freight cars, to make a train extending almost to the city of Philadelphia. The growth of only a quarter of a century has made Chicago the largest original grain market in the world, not excepting Odessa, the famous grain market on the Black Sea.

From whence comes this prosperity—this rapid growth? It may be all told in one word, and in it is taught us an important lesson—*Enterprise*

To Chicago, then, as the metropolis of Illinois, and to Illinois, the leading State of the great North-West, I again bid you welcome. The soil on which you stand to-day is too new to boast of ancient or classic memories, yet it is rich in the most sacred and patriotic associations. It is the home of statesmen, heroes and patriots, that have stood forth nobly in defence of the institutions founded by our fathers, and whose fame has gone forth over the whole world.

In the suburbs of our city, on the shore of Lake Michigan, you will stand by the grave of the gifted and patriotic Douglas, whose eloquence once thrilled our halls of legislation, and who, dying just as the greatest rebellion the world has ever known, was bursting upon the nation, left us an inheritance to his children, and to his countrymen a legacy, which so long as time shall last, shall stand out brightly on the page of history, the immmortal words, "Obey the laws and support the constitution of your country."

And here, too, in his quiet Illinois home, engaged in the duties of civil life, was found the great soldier and military leader, whose genius and heroism, after carrying the armies of the Republic through the bloody battles and victories of Donelson, Shiloh, Vicksburg, Chattanooga, Spotsylvania and Richmond, at last won for us an honorable and lasting peace, and made the name of Grant immortal as is the history of his country, and of whom it may be said, as of one of England's great leaders, "he never fought a battle that he did not win, and never encamped before a city that he did not take."

And finally, in this city, five years ago last month, in a building erected for the purpose, and which is still standing, was placed in nomination for the Presidency, Illinois' honest and cherished son, whose sagacity and statesmanship guided our country safely through the perils of a gigantic rebellion, and whose untimely death left a nation in tears. Millions yet unborn, will tread these streets, as they wend their way over the Western prairies, on their pilgrimage to the grave of Liberty's noblest martyr, ABRAHAM LINCOLN.

Again, gentlemen, I bid you welcome. Welcome to your duties—welcome to your pleasures—welcome to Chicago.

### STEAM GAUGES FOR VULCANIZERS.

BY GAM'L JACKSON.

In the December number of the REGISTER is an article on this subject, which is amplified by the same writer in the issue for March and April.

The first article very properly discusses the power and danger of steam under high pressure, and makes a valuable suggestion regarding the strength that vulcanizers should possess.

I happened to be vividly awakened on this subject about three years ago by the ascent of the top of my old fashioned vulcanizer through the ceiling of my laboratory. The accident occurred just as they all occur, from *forgetting the apparatus*, and would not have been averted a jot by the use of an "$18" steam gauge.

The only practical difference between a thermometer and a gauge is that the indications of heat and pressure (which are here synomymous terms) are seen with less effort on the gauge on account of the superior size of the scale. Neither alone is at all safe. The thermometer is safer because it is of remarkably simple construction, whilst the gauge is comparatively a complicated machine, with spring, cog-work and piston. The gauge must be watched just as closely as the thermometer, and is more liable to be forgotten on account of the "refreshing security" attending its use. This security is of that delusive kind which a superficial man would feel in looking through a reversed field-glass at a battery of "100 pound Parrott guns" pointed at him at short range. The feeling of "refreshing security" may, however, in a certain instance, arise from familiarity with "a small steam engine" employed in the manufacture of "Adamantine, Self-Hardening Platinum Cement," vulgarly called amalgam.

The safest and cheapest method of vulcanizing is to use a thermometer and a safety valve. The thermometer gives uniform and satisfactory results, and the valve in conjunction is reliable protection against danger.

I use in preference to all others the old-fashioned three flask vulcanizer, described in Richardson's Mechanical Dentistry, with the thermometer extending through the top. The safety valve is made of brass, and consists of a hollow cylinder $\frac{3}{4}$ of an inch in diameter, extending $1\frac{1}{2}$ inches above the top of the vulcanizer. Its inner diameter is $\frac{1}{2}$ an inch. A cap $\frac{3}{4}$ of an inch deep fits loosely on the top, and contains a gasket of rubber packing. A well constructed hinged lever is hung in the usual way, so as to bear on an elevation at the center of the cap. The power consists of weights on a platform suspended 6 inches from the resistance. The distance from the resistance to the fulcrum is ½ an inch. If well made the valve will remain closed at 320° and will open at 325°.

I constructed the valve I use, and presume that there are none like it, with respect to the joint, for sale; but S. S. White gives a cut of an "Improved Copper-Boiler Vulcanizer" in the advertisements of the *Cosmos*, which altogether appears to be constructed upon correct principles. I presume, however, that the safety valve has a metallic ground joint; if so, I think it would be improved by substituting rubber packing as above described.

In using new rubber gaskets, chalk alone should be rubbed on the parts to be separated, for a few times, after which nothing will be required; and if the top is put on always the same way, no leakage will occur.

Having thus a vulcanizing apparatus perfectly *effective, simple* and *safe*, I am at a loss to understand the desire for further improvement in this direction, and would recommend bomb-proofs to dentists who rely upon steam gauges alone for safety.

